# Tubular cell loss in early *inv/nphp2* mutant kidneys represents a possible homeostatic mechanism in cortical tubular formation

**DOI:** 10.1371/journal.pone.0198580

**Published:** 2018-06-11

**Authors:** Masaki Shigeta, Hirotaka Kanazawa, Takahiko Yokoyama

**Affiliations:** Department of Anatomy and Developmental Biology, Graduate School of Medicine, Kyoto Prefectural of Medicine, Kyoto, Japan; Anatomy, SWITZERLAND

## Abstract

Inversion of embryonic turning (*inv*) cystic mice develop multiple renal cysts and are a model for type II nephronophthisis (NPHP2). The defect of planar cell polarity (PCP) by oriented cell division was proposed as the underlying cellular phenotype, while abnormal cell proliferation and apoptosis occur in some polycystic kidney disease models. However, how these cystogenic phenotypes are linked and what is most critical for cystogenesis remain largely unknown. In particular, in early cortical cytogenesis in the *inv* mutant cystic model, it remains uncertain whether the increased proliferation index results from changes in cell cycle length or cell fate determination. To address tubular cell kinetics, doubling time and total number of tubular cells, as well as amount of genomic DNA (gDNA), were measured in mutant and normal control kidneys. Despite a significantly higher bromodeoxyuridine (BrdU)-proliferation index in the mutant, total tubular cell number and doubling time were unaffected. Unexpectedly, the mutant had tubular cell loss, characterized by a temporal decrease in tubular cells incorporating 5-ethynyl-2´-deoxyuridine (EdU) and significantly increased nuclear debris. Based on current data we established a new multi-population shift model in postnatal renal development, indicating that a few restricted tubular cell populations contribute to cortical tubular formation. As in the *inv* mutant phenotype, the model simulation revealed a large population of tubular cells with rapid cell cycling and tubular cell loss. The proposed cellular kinetics suggest not only the underlying mechanism of the *inv* mutant phenotype but also a possible renal homeostatic mechanism for tubule formation.

## Introduction

Inversion of embryonic turning (*inv)* mutant mice develop situs inversus, jaundice and polycystic kidneys, with most mutants dying before postnatal day (P) 7 [1, 2]. Subsequently, mutations in *inv* were identified as being responsible for human type II nephronophthisis (NPHP2), an infantile autosomal recessive renal disorder [3]. The cystic phenotype, including tubular dilatation, was shown to be similar in mice and humans [4]. The C-terminal domain of *inv* is poorly conserved in mice and humans, while the N-terminal domain with ankyrin repeats is highly conserved [5]. The C-terminal domain of the mouse *inv* was reported to be important for its interaction with the serine-threonine kinase Akt, which plays important roles in cell survival [6]. Introduction of a modified *inv* gene, lacking the C-terminus (*inv*DC), rescued all *inv* phenotypes except for cystic kidneys [7, 8]. Because the *inv*DC mutant survives longer than *inv* mutant, the *inv*DC model is useful for investigating renal cystogenesis, excluding its other associated abnormalities.

Cystogenesis in polycystic kidney disease (PKD) is the most typical phenotype in ciliopathy [9] because the responsible proteins, such as the polycystin-1 and polycystin-2, are localized in cilia of renal epithelia [10] [11]. In models for autosomal dominant polycystic kidney disease (ADPKD), abnormal proliferation was considered to be one major phenotype, along with epithelial simplification and other phenotypic changes, such as apoptosis, that are likely to be important [12] [13] [14]. We previously showed that *inv*DC cystic kidneys had significantly increased cell proliferation and apoptosis in the later, severe stages of cystogenesis [8]. However, the tubular cell kinetics in postnatal early cystogenesis with the *inv* mutation remains unknown. In addition, it is generally unclear how the abnormal proliferation is linked to cell death, such as apoptosis, in PKD and its biological significance has not been well addressed.

Although pathogenic cellular phenotypes, such as oriented mitotic defect, are associated with the collecting duct in the renal medulla [15] [16], the underlying cellular kinetics in the cortical cystogenesis observed in NPHP models such as in *inv*DC mutant remains unexplained. Notably, most cortical tubular cells in these models appeared to have a slow cell cycle with final cell division occurring postnatally, an effect characterized by rapid growth decline from P15 to P30 [17]. This finding was different from those in *in vitro* cell lines, because these continued to proliferate. Studies using conditional knockout mice showed that severity or onset of the polycystic phenotype occurred within the developmental window of up to P14 [18, 19]. These observations raised intriguing questions about the role of increased proliferation in early cortical cystogenesis with the *inv* mutation, that is, it is still uncertain whether the abnormal cell proliferation was caused by changes in cell cycle length or a defect in growth control in the kidneys *in vivo*.

The purpose of our study was to investigate the significance of proliferation in early cortical cystogenesis and to clarify the complicated cellular kinetics using mathematical modeling. We showed that total tubular cell numbers were unaffected in early cortical cystogenesis, despite a significant increase in the proliferation index, measured by bromodeoxyuridine (BrdU) labeling. Unexpectedly, tubular cell loss with abnormal nuclear protrusion and debris was identified as an early cystogenic phenotype in the *inv*DC mutant although no typical apoptotic cell death was detected. Based on current experimental data, we established a new multi-population shift model involving a large population of tubular cells with rapid cell cycling, along with increased tubular cell loss in a population with slow cell cycling. The proposed tubular cell kinetics model demonstrated the critical role of tubular cell loss in both renal tubular cystogenesis and homeostasis.

## Results

### Tubular cell number was unaffected in early *inv*DC kidneys despite an elevated BrdU proliferation index

*Inv*DC kidneys show normal morphology until P15, while early cystic tubules begin to emerge spontaneously within the cortex beginning from P7 [8]. Therefore, we characterized early cystogenesis from P9 to P15 to determine the cellular basis for the increased cell proliferation index we observed. Although the proliferation index was significantly increased in the *inv*DC cystic model (Table 1 and S1 Fig), overall tubular growth in the postnatal cortex showed a similar pattern to that of kidneys in control mice. This implied that a homeostatic mechanism maintaining (or controlling) cell number was active, even in the *inv*DC mutant cystic model.

**Table 1:** Double proliferation indexes in tubular cells from control and *inv*DC renal cortices.

	BrdU (labeling at 3 hr before sampling)		EdU (labeling at 24 hr before sampling)	
	control	*inv*DC	control	*inv*DC
**P4**	**0.1% (1/1556)**	**2.2% (53/2373)**	**1.3% (21/1556)**	**5.9% (133/2373)**
**P9**	**4.3% (128/2947)**	**5.6% (143/2557)**	**9.0% (265/2947)**	**12.4% (317/2557)**
**P15**	**3.3% (103/3158)**	**5.8% (147/2521)**	**9.8% (309/3158)**	**15.3% (386/2521)**

EdU was intraperitoneally injected into control and *inv*DC mutant mice at 24 h before kidney sampling at the indicated postnatal days (P), followed by the intraperitoneal administration of BrdU after 21 h. Kidney sections were processed for BrdU- and EdU-staining as described in Materials and Methods. The proliferation index (%) was respectively calculated by dividing the number of BrdU- or EdU-positive tubular cells by the total tubular cell number which is accumulated from 3 or 4 mice in each group (n ≥ 8 imaging fields per mice), and columns indicate the groups of mice the sections were from. The differences in both BrdU- and EdU-based proliferation rates were statistically significant between control and *inv*DC mutant cells (*P* < 0.05; Fisher`s Chi-squared test). Note that the double-labeling ratio, which indicates the proportion of cells re-entering S phase, was barely detectable in both control and mutant cells with this time lag. The scarce amount of BrdU labeling in tubular cells at P4 was consistent with data shown in S1 Fig.

**Fig 1:**
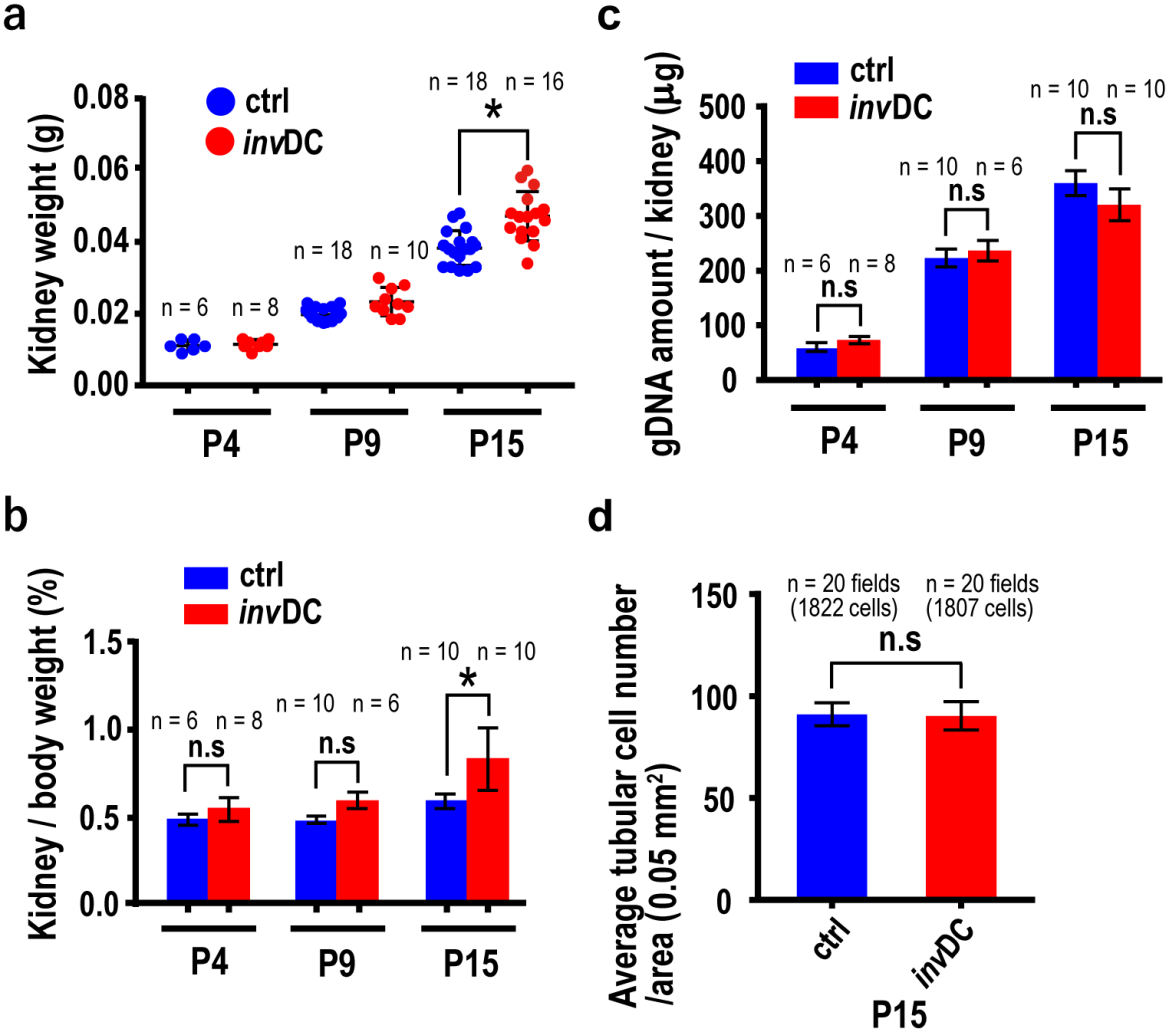
Early cystogenesis in *inv*DC mutant kidneys at P15 showed no significant increase in cortical tubular cell number, compared with in control kidneys. (**a**) Comparison of kidney weight between control and mutant mice (*P* < 0.05, Kolmogorov-Smirnov test). **(b)** Comparison of kidney/body weight ratio between control and mutant mice. **(c)** There was no significant difference in the amount of whole kidney gDNA between control and mutant mice. **(d)** Comparison of average tubular cell number per cortical area between control and mutant mice at P15 (± SEM). The Mann-Whitney U test was used, with *P* < 0.05, in panels **(b) (c)** and **(d)**.

To understand if the proliferation index, based on BrdU-labeling, was linked to the resulting cell number increases in *inv*DC kidneys, we compared the weight, genomic DNA (gDNA) levels and tubular cell number per renal cortical area in mutant and control kidneys. Kidney weights or kidney/body weight ratio in mutant mice were not significantly increased at P4 or P9, compared with in controls (Fig 1a and 1b). Although kidney weights in mutant mice began to increase significantly from P15, these values were potentially overestimated because of increased cystic fluid in the spontaneous cystic tubules. Consistent with this, dissected kidneys had lower weights. There were also no significant differences between mutant and control mice in levels of gDNA in the whole kidneys (Fig 1c). The gDNA content was significantly decreased in the later, severely cystic kidneys (at P30), compared with in controls (S2 Fig). This was consistent with previous reports that apoptosis was significantly elevated at the later stage of kidney damage [8] [20]. Furthermore, average tubular cell number per cortical area was not significantly different in the two mouse strains (Fig 1d). Taken together, these results indicated that the elevated proliferation index was not accompanied by an increase in total tubular cell number in the early *inv*DC kidneys.

**Fig 2:**
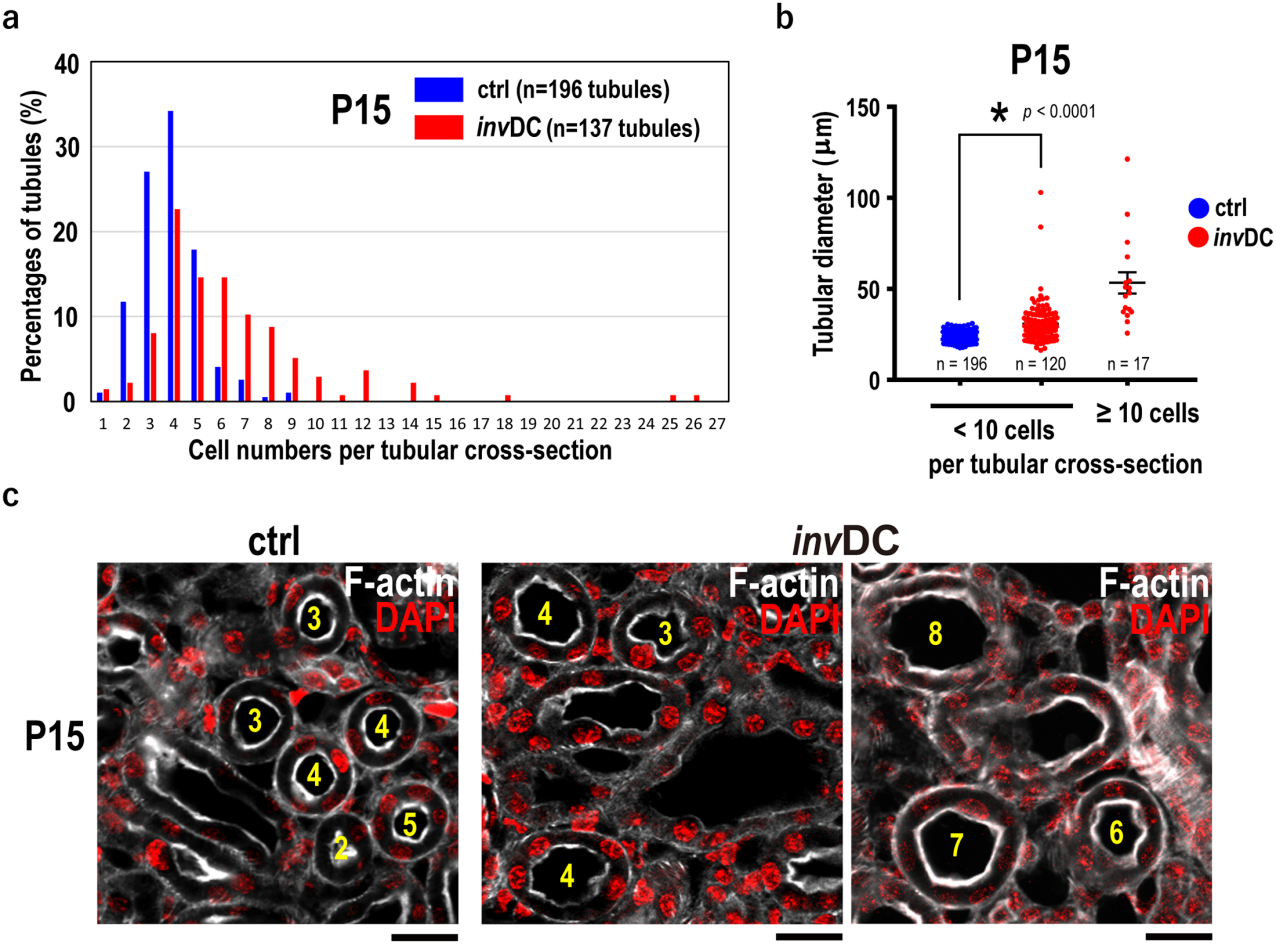
Early *inv*DC mutant cortex exhibits tubular dilatation without an increase in cell numbers as well as increased cystic tubules with a high cell number. **(a)** Histogram showing estimated percentages of the tubules having the indicated cell numbers per tubular cross-section. The *inv*DC mutant at P15 had increased percentages of tubules with a high cell number per tubular cross-section compared to the values obtained from controls. Note that total number of cross-sectional tubules was decreased in mutant (n = 137) relative to control (n = 196) with the same cortical area. **(b)** Comparison of the relationship between tubular diameter and cell number in control and mutant mice (± SEM). The Mann-Whitney U test was used, with *P < 0.05. **(c)** Images of cortical tubular morphology merged with F-actin (white) and DAPI (red) (left, control; middle and right, mutant). It is noticed that yellow numbers indicate tubular cell numbers per each tubule cross-section, clearly showing that a tubular dilating was occurred in *inv*DC mutant tubules of same cell number with controls. Scale bar, 10 μm.

Although cystic tubule is morphologically characterized by the increased cell number and/or tubular dilation, these phenotypes are not fully quantified in early *inv*DC cystogenesis. To evaluate the relationship between tubular cell number and diameter, we first performed the histogram analysis of the data in Fig 1d focusing on cell counts per tubular cross section. The histogram analysis showed that the percentage of tubules having from 2 to 4 cells was higher in control mice than in mutants, while the percentage of tubules having more than five cells was higher in mutants than in the controls (Fig 2a). It is noteworthy that the percentages of cystic tubules were increased in mutant, while the total tubule number was decreased (n = 137) relative to control (n = 196) with the same cortical area. The average cell numbers accumulated from all tubules per area (n = 20) were also not statistically significant (43.7 ± SEM = 4.1 in control; 38.0 ± SEM = 3.3 in mutant) even when selecting the cross-sectioned tubules, which was compatible with the result of Fig 1d. Interestingly, we found that the tubular diameter in *inv* mutants was larger than in the controls when tubules with the same numbers of cells (< 10 cells per tubule) were compared (Fig 2b). This implies that tubular dilatation occurred without an increase in cell numbers per tubular cross-section as the initial process during early cytogenesis and, subsequently, the enlarged cystic tubules were linked to the apparent cell number increase.

### Temporal doubling time analysis of tubular cells during early cortical cystogenesis

**Fig 3:**
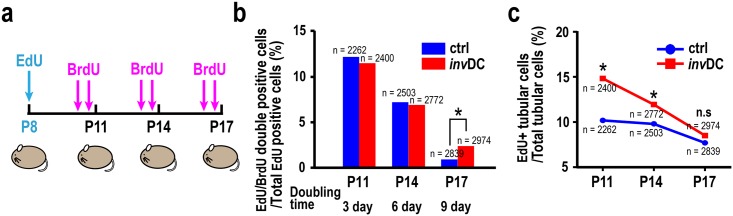
Decreased number of EdU-labeled tubular cells indicated possible tubular loss during early cystogenesis in *inv*DC kidneys. **(a)** Scheme of the EdU/BrdU double-labeling experiment. EdU was injected intraperitoneally into the mice at P8, followed by multiple BrdU injections at 6 h and 12 h before the indicated harvesting days to increase the detectability of double-labeled cells. After isolation of kidneys, EdU and BrdU staining were performed sequentially as described in Materials and Methods. Tubular cells in the renal cortex of mice with each age (n = 3) were evaluated for double labeling experiments. **(b)** Quantitative data showing that there was no overall shift in cell cycle pattern during postnatal renal development between control and mutant mice at P11 and P14. It is noticed that the percentage of EdU/BrdU double positive cells was significantly elevated in mutant at P17. **(c)** Time-dependent decline of the percentages of EdU-positive tubular cells per total tubular cells in the early cystic kidneys. (*P < 0.05, Fisher’s Chi-squared test).

Increases in the BrdU-based proliferation index can reflect changes in one entire cell cycle length. Therefore, we next compared the doubling times of tubular cells in the controls and mutants. This was evaluated by determining entry into the second S phase (re-entry into next cell cycle) based on the principle that both 5-ethynyl-2´-deoxyuridine (EdU) and BrdU can be incorporated the cells in S phase. Thus, the double labeling experiment was performed, referred to previous report using kidneys [17] and the percentages of double positive cells per total tubular cells were then compared at the indicated time points (Fig 3a). Unexpectedly, there was no change in doubling times in mutant cells at P11 and P14, although the population doubling time at P17 was significantly higher in the mutants than in the controls (Fig 3b). This may have indicated either a minor population of cells re-entering the cell cycle or a changed S phase length. Because the length of the S phase in the *inv*DC mutant was comparable to that in the controls at P15 (S3 Fig), we concluded that the doubling times were comparable in early polycystic kidneys of the mutants and in normal kidneys of control mice, at least until P15. Taken together, considering that total cell numbers were no different in control and mutant kidneys (Fig 1), these results suggested that tubular cell loss likely occurred in renal cystogenesis, preventing an overall increase in cell number. This was demonstrated by temporally tracing the percentages of EdU-labeled tubular cells per total tubular cells (Fig 3c). The elevated EdU-labeling ratio in P8 mutant mice was decreased over time to the same extent as in controls, until P17.

### Establishment of a multi-population shift model for tubular cell kinetics in renal tubule formation

**Fig 4:**
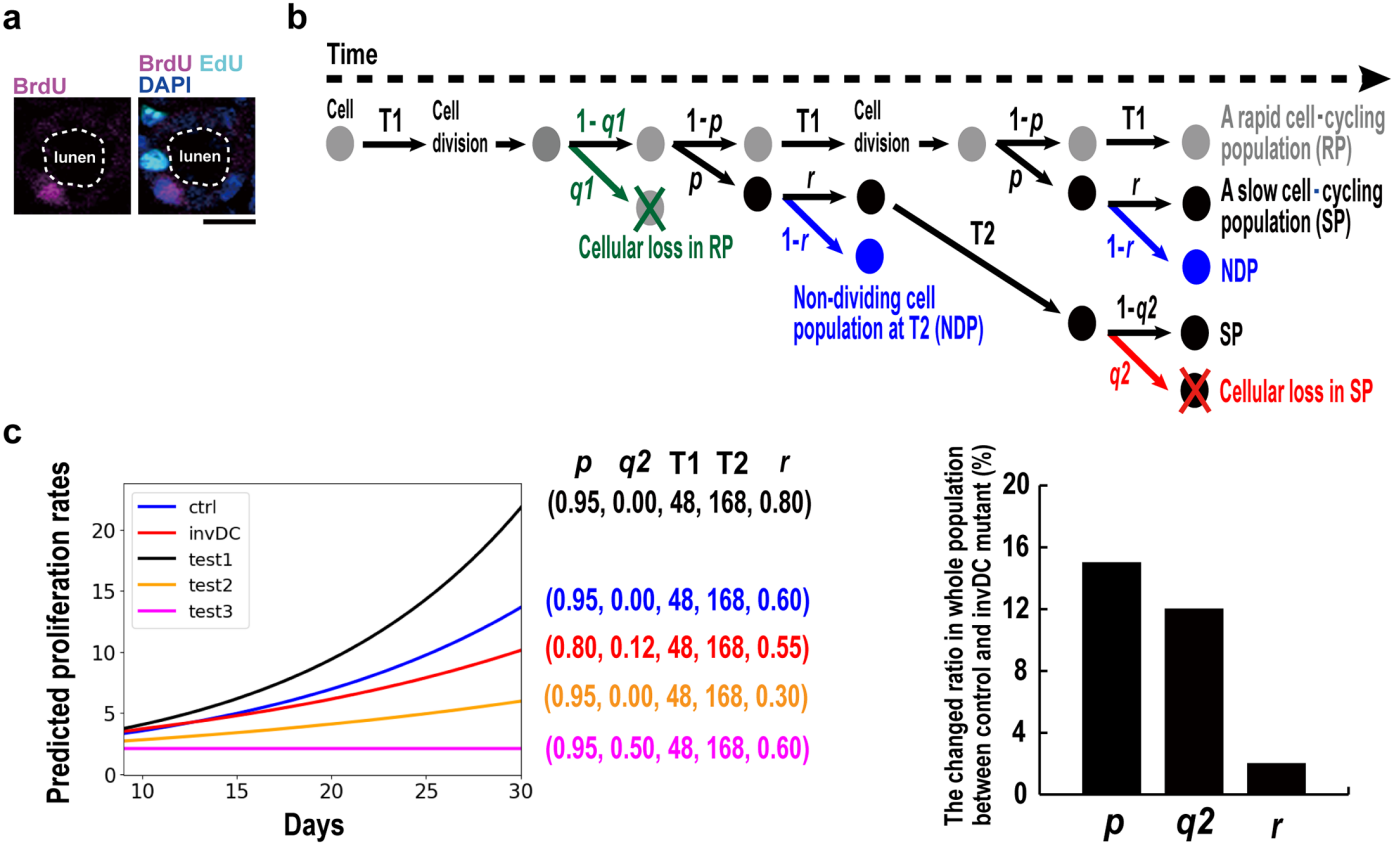
A mathematical model revealed both a large population of tubular cells with rapid cell cycling and cell loss in a slow cell cycling population during early cystogenesis (P9–P15). (a) EdU (24 h before)- and BrdU (3 h before)-labeled cells adjacently resided in the identical cross-sectioned tubule, suggesting that the adjacent cells including divided daughter cells re-entered cell-cycling with a different timing. (b) Hypothetical proliferation model of a multipopulation with probability of cellular loss in both RP (gray) and SP (black) and cell division in SP. All mathematical parameters used are described in Table 2. (c) Mathematical simulation between the predicted proliferation rates and developmental days from P9 to P30 under the condition combined with various indicated probabilities from the initial population with 1 at day 0. Control and *inv*DC proliferative phenotype best fitting with experimental proliferation rates values at P9 and P15 (Fig 1b) and P30 (S2 Fig) are indicated as blue and red line, respectively.

**Fig 5:**
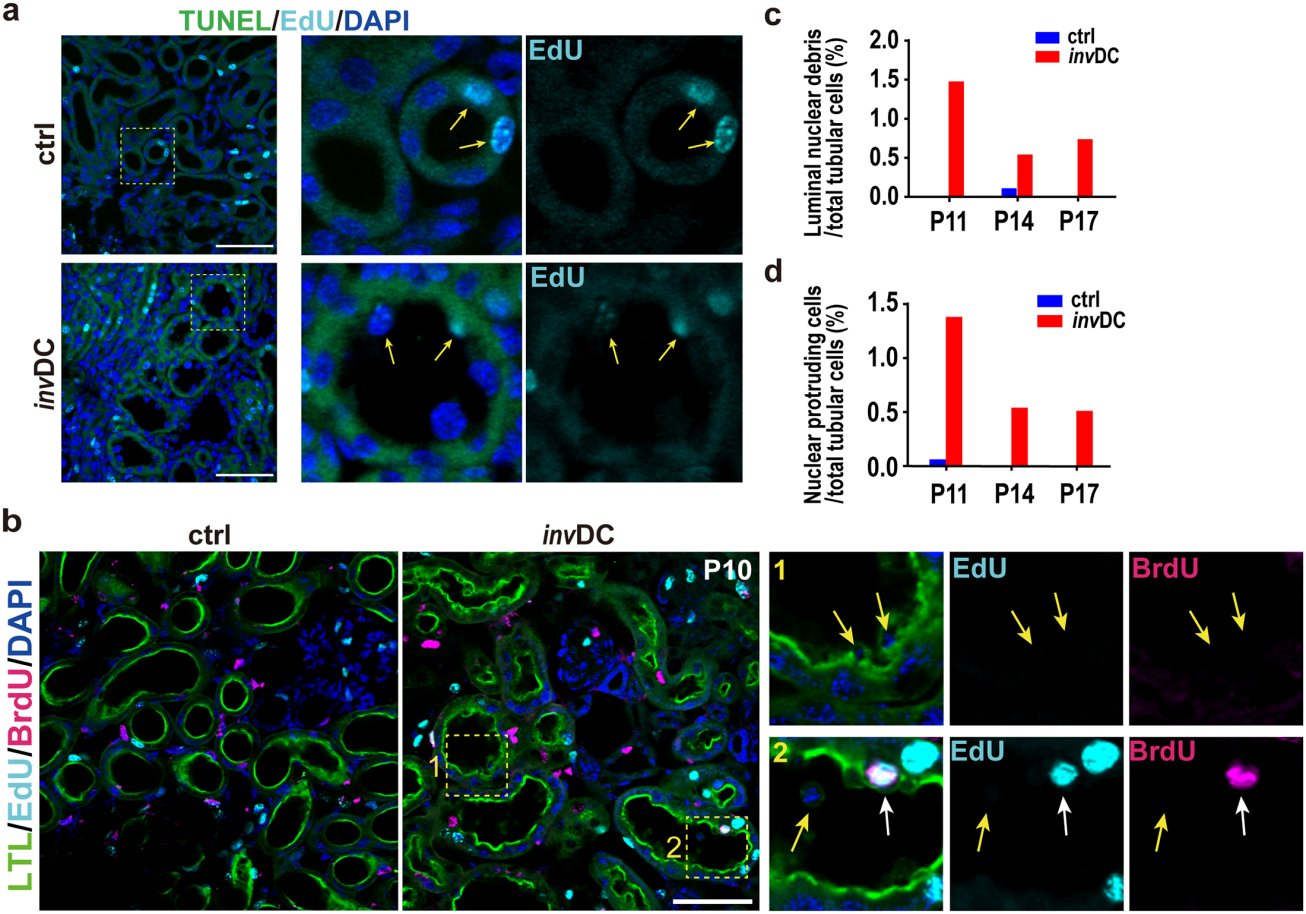
Luminal nuclear protrusion and debris were linked to early cortical cystogenesis in *inv*DC kidneys. **(a)** There were no TUNEL-positive (FITC green) tubular cells in the cortex at P15. Nuclei with incorporated EdU (cyan) (yellow arrows) and DAPI staining (blue) were visualized. **(b)** Luminal nuclear debris (yellow arrows) and protruding cells (white arrows) in tubules during early cystogenesis at P10. Incorporated EdU (cyan) or BrdU (magenta) was visualized with nuclear staining with DAPI (blue) and LTL (green). The EdU/BrdU double-labeled cells (white arrows) were often observed with apical nuclear protrusions. **(c)** The percentages of luminal debris or (**d**) the percentages of cells with apical nuclear protrusions per total tubular cells at the indicated postnatal mice age (control: n = 1721 at P11, n = 1795 at P14, n = 2200 at P17; *inv*DC mutant: n = 1886 at P11, n = 1866 at P14, n = 2155 at P17). *p <* 0.01 in comparison to the all control group are considered significant using a Fisher’s Chi-square test.

To analyze the cellular kinetics in early cystogenesis, we established a mathematical model for tubular development on the basis of current data and other observations. First, we found that BrdU-positive tubular cells (3 h before labeling) were always detectable in an isolated pattern in each cross-sectioned tubule and that double positive cells were never detected in the same tubule in both controls and *inv*DC mutants (Fig 4a). In contrast, the EdU-labeling experiment (24 h before labeling) exhibited paired EdU-positive cells with adjacent labeled cells first appearing within 24 h. This indicated that the EdU-incorporating cells each divided into paired daughter cells (refer to Fig 5a) because renal tubular cells are thought not to migrate far. Collectively, these observations suggested that each adjacent tubular cell did not divide at the same time, as supported by the fact that cells positive for EdU (24 h before labeling) and BrdU (3 h before labeling) resided next to one another in identical tubules (Fig 4a). We reasoned that heterogeneous tubular cell populations, as suggested in Fig 3b, were derived from an original population dividing in an asymmetrical mitotic manner. Based on this concept, we designed a multi-population model (Fig 4b), utilizing a shift probability (*p*) from a rapid cell cycling population (RP) to a slow cell cycling population (SP) as incidence rate, giving rise to asymmetrical mitosis. The doubling time of each population was defined as T1 = 2 d for SP and T2 = 7 d for RP, based on the following considerations: (1) there are peak population doubling times (3 and 6 d) in postnatal renal development (Fig 3b) and (2) this doubling time pattern was similar to that observed in a previous study, using double labeling with the BrdU and ^3^H-thymidine, showing that there were two major peak population doubling times in cortical tubular cells (2–3 and 6–7 d) [17]. Considering that there was tubular cell loss the in *inv*DC mutants (Fig 3c), the probability of cellular loss *q*1 in RP and *q*2 in SP was introduced into the model. For the mathematical model fitting, values for average growth rate (P15/P9) and (P30/P15), calculated as the rates of increase in whole kidney gDNA, were utilized (Fig 1b and S2 Fig). This was based on the criterion that total tubular cell number was comparable in controls and mutants until P15 (Fig 1). In the simulations, we found that cellular loss in SP, but not RP, was a better fitting model for the *inv*DC mutant phenotype and this finding was consistent with the observation that the increased tubular cell numbers were beginning to decrease from P11 (Fig 3c). However, the model still did not sufficiently explain the growth decline from P15 to P30 in later cystic kidneys, accompanied by a significant decrease in whole kidney gDNA (S2 Fig). Importantly, we noticed that it was necessary to also introduce the probability of re-entering cell cycle (*r)* at 168 h (T2) in SP for the model to be consistent with a significantly decreased gDNA at P30. In this context, the probability, *r*, must be 0.60 in the controls (Fig 3c, blue line: *p* = 0.95, *q*2 = 0.00, *r* = 0.60) to fit the experimental values for change in average growth ratios from P9 to P30. The non-dividing cell population with “1-*r*” (NDP) biologically indicated both a quiescent population and a slower cell cycling one at > T2, two possibilities that would be difficult to distinguish experimentally. Among the various simulation values, we identified the best fitting model for the *inv*DC mutant phenotype (Fig 4c, red line), demonstrating that a large population of tubular cells with rapid cell cycling (*p* = 0.95 → 0.80) and tubular cell loss (*q*2 = 0.00 → 0.12) were accompanied by another population that had exited the cell cycle (*r* = 0.60 → 0.55). The proposed model showed that both cell proliferation and cell loss occurred, explaining why cell numbers appeared to be unaffected in *inv*DC mutant mice until early cystogenesis at P15 (Fig 1). That is, under conditions of cell loss, the shift probability (*p*, 0.95 → 0.80) can decrease if the whole population size is equal to that in a model with no cell loss. Therefore, the increased BrdU or EdU proliferation indexes can be interpreted as an increase in the RP by the decreased shift to the SP, suggesting a defect in cell fate decision (control of the probability shift) rather than in cell cycle machinery. Taken together, the experimental findings and the mathematical model demonstrated a renal homeostatic mechanism in which proliferating tubular cells in RP of the *inv*DC mutant are cleared by cell loss in SP. Although the model also implied a population re-entering cell cycle at T2 in both controls (*r*, 0.60) and mutants (*r*, 0.55), the ratio of change in the whole population was approximately 2.0% (Fig 4c right graph). Therefore, we concluded that tubular cell loss following uncontrolled proliferation (*q*2, ctrl: 0.00 → mutant: 0.12) was more critical in early *inv*DC cystogenesis.

### Significant tubular cell loss and γH2AX signaling in early cortical cystogenesis

**Fig 6:**
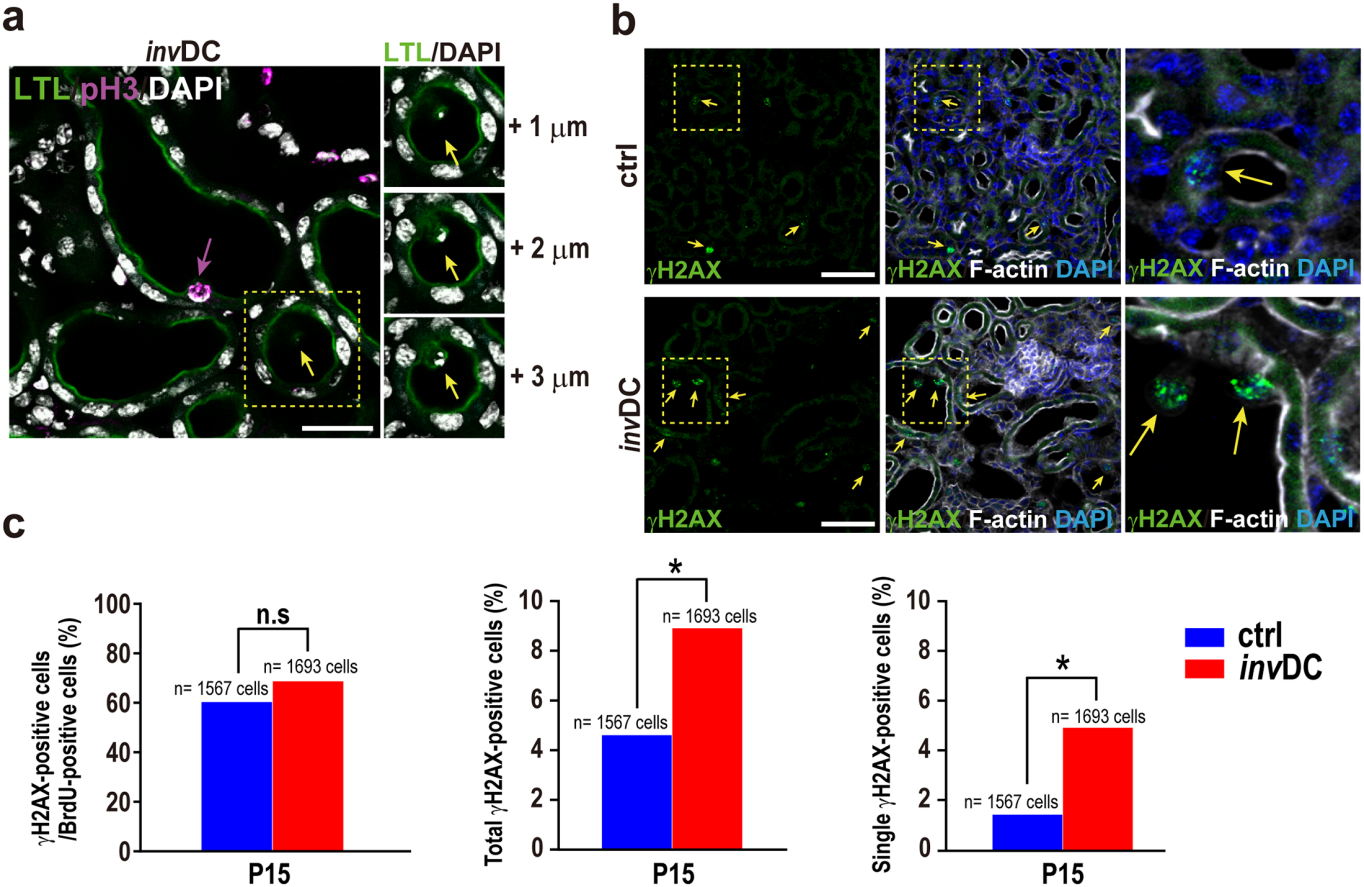
Expression of pH3 and γH2AX in nuclear protrusions. **(a)** Luminal nuclear debris bound to precystic tubules in *inv*DC mutant at P15 (light inset). The nuclear protrusions were predominantly pH3-positive (magenta). (**b**) γH2AX expression in nuclear protrusions or sloughing cells with a dotted staining pattern in mutant tubular cells. Right column images were merged with F-actin (white) and DAPI (blue) staining. Scale bar, 20 μm (white). **(c)** γH2AX is a marker of S/G2 phase cell cycle in both controls and mutant renal tubules (left graph). Significant difference of total or single γH2AX expression ratio between controls and mutants (middle and right graph). *P < 0.05, Fisher’s Chi-squared test.

To investigate apoptosis as a possible cause of cellular loss, we performed terminal deoxynucleotidyl transferase dUTP nick end labeling (TUNEL). TUNEL-positive apoptotic tubular cells were not detected in early cystogenesis at P15 in the renal cortex (Fig 5a), while a TUNEL signal was clearly detected in controls after DNase I treatment or in cisplatin-induced apoptosis of the renal cortex (S4a–S4d Fig). However, we observed increased luminal nuclear debris of various sizes and with irregular shapes in mutant mice (Fig 5a and 5b). Striking nuclear protrusions or sloughing cells were also observed in the tubular lumens and some of the cells had brush border loss (Fig 5b–5d). These cellular phenotypes closely resembled those of acute tubular necrosis (ATN) [21], but appeared to be more cell cycle-dependent because most of the protruding cells expressed the G2/M phase marker, phospho-histone H3 (pH3) (Fig 6a). Although the sloughing tubular cells appeared to be dropped into the tubular lumens (a classical observation in ATN), we noticed that they were bound to the luminal side. Thus, this tubular phenotype suggested cell loss in early cystogenesis and was likely to involve renal phagocytic clearance by adjacent tubular cells, as recently described [22]. In support of this, we observed luminal binding of isolated luminal debris, even in precystic tubules (Fig 6a inset).

The DNA damage response (DDR) was recently analyzed in models of PKD [23, 24], while γH2AX, a molecular marker for the DDR, was implicated as a non-apoptotic marker of mitotic cell death [25]. Thus, γH2AX expression is considered by some to indicate a specific form of cell death [26, 27]. Interestingly, a recent *in vivo* study using the gut, a tubular organ, also showed mitotic catastrophe with γH2AX-positive but TUNEL-negative cells involved in gut homeostasis [28]. We observed γH2AX expression in the nuclei of luminal sloughing cells in the *inv*DC mutants (Fig 6b) in addition to the BrdU-positive cells in both mutants and controls (Fig 6c, left), the latter regarded as a cell cycle marker from the middle stage of S phase to G2 or M [29] [30]. We found that total γH2AX expression in the *inv*DC mutant (8.9%) was higher than in controls (4.6%), while the BrdU-negative and γH2AX-positive cell populations were also significantly elevated (control, 1.4%; *inv*DC, 4.9%) (Fig 6c, middle and right). In the controls, the BrdU-negative and γH2AX-positive cell population was likely to represent those retained in the G2 phase because BrdU was injected at 3 h before harvesting of the kidneys. If the average BrdU-labeling ratio (mutants/controls = 1.5) from P9 to P15 (Table 1) was proportionally applied to a possible ratio estimation of the G2 phase population, the single γH2AX-positive pool (4.9%) must have contained both the additional cells (2.8%), possibly with damaged DNA, and G2-retained cells (2.1%). Overall, elevated γH2AX-expression in the *inv*DC mutants suggested signs of a possible mitosis-linked cell death and DNA-damaged cells exited from the cell cycle, in addition to an increased BrdU-labeling index.

## Discussion

### Active role of tubular cell loss in renal tubular hemostasis

Control of organ size is mediated by the balance of cell proliferation, differentiation and apoptosis in multicellular organisms [31]. Some evidence in PKD models suggested that both increased cell proliferation and apoptosis occurred in polycystic kidney tubules [12] [13] [14]. Our previous study using the *inv*DC model showed significant apoptosis in the later, severe stage of cystogenesis, while TUNEL-positive cells were detected during early cystogenesis, at P7 [8]. However, the cell number-based, not tubule-based, ratios were very low (0.010% in controls; 0.016% in mutants). These previous results could also have been explained by accidental cell death with an allowable limit of error and, therefore, did not seem to be a significant phenotype, at least in early cystogenesis.

In the present study, we focused on tubular cell kinetics during early cystogenesis, up to P15, in an NPHP model and re-evaluated renal tubular proliferation dynamics. Unexpectedly we found that tubular cell loss, possibly non-apoptotic cell death, as indicated by γH2AX expression, was a critical phenotype in early *inv*DC cystogenesis. We could not identify the type of cell death because there were no distinct markers for necrosis (or controlled necrosis, known as necroptosis) or apoptosis. However, we did observe nuclear debris in the renal tubules in the *inv*DC mutants, connected to adjacent tubular cells. This suggested that the dying tubular cells were rapidly cleared, through phagocytosis by adjacent tubular cells [22]. Thus, proliferating cells were likely cleared by phagocytosis to maintain both cell number and tubular monolayer integrity. To precisely quantify this type of cell death, further experiments using the *inv*DC mutant mated with a multicolor and multiclonal rainbow mice [32] might be appropriate, enabling phagocytosis-mediated clearance to be quantified by overlapping staining colors.

Deletion of the anti-apoptosis gene, Bcl-2, resulted in polycystic kidney formation (similar to PKD) [33], demonstrating that controlled tubular cell death may play an active role in physiological renal tubular formation. In the Bcl-2 deficient kidney, significant numbers of pyknotic nuclei (an indicator of cell death) were observed in the interstitium, rather than in tubular cells [34]. This suggested that non-apoptotic cell death can be occurred in cortical tubular cells lacking Bcl-2, because Bcl-2 is also associated with necrosis and autophagy [33]. Given that phagocytosis-mediated clearance of tubular cells occurred during tubular formation in early *inv*DC cystogenesis, apoptotic signaling might be abolished or masked by unknown phagocytotic process. Then, at the later stage of cystogenesis, tubular phagocytotic capacity might decline or be fully inhibited and, thereby, striking elevations of apoptosis might be detectable. The gDNA content in the kidneys of dying *inv*DC mutants, at P30, was significantly lower than in the controls. This was consistent with an increase in apoptosis at the later stage, as we previously described [8]. Similarly, late stage apoptosis was also reported during cyst regression in a PKD model [20]. In these types of cystic kidney models, the balance between cell proliferation and cell loss can differ depending on the stage of disease progression. A significant aspect of our study is that the postulated cellular kinetics behavior was demonstrated by mathematical modeling, enabling a clear understanding of the relationship between abnormal cell proliferation and cell loss during cystogenesis.

### Biological significance of γH2AX signaling in the *inv*DC polycystic kidney model

The DDR signaling marker, γH2AX, was markedly increased in some polycystic kidney models [23, 24] and one of the genes involved in DDR, NEK8, appeared to work through downstream signaling in the *inv* mutant model [35]. Consistent with this, we detected increased γH2AX expression in the *inv*DC mutant renal cortex, with most expression in the S phase cell population in the controls. It is noteworthy that γH2AX expression was detected in abnormal protruding cells in *inv*DC tubular lumens. Because apical mitosis occurred with interkinetic nuclear migration (INM) in renal ureteric tubules, abnormal tubular protrusion may imply a process predicting mitotic cell death [25]. Importantly, previous studies showed that loss of *inv/nphp*2 cause tubular cell hypertrophy and increased bi and multinucleated cells [4, 36]. These phenotypes might be derived from mitotic catastrophe marked with γH2AX, which we speculate in *inv*DC mutant, because it also can exhibit abnormal mitosis resulting in aneuploidy or multinucleation in addition to mitotic cell death [37].

Interestingly, γH2AX can be detected as a ring-like staining pattern around the nucleus in early apoptotic cells, a pattern distinguishable from the dotted staining pattern observed during the DDR [38]. We observed only the dotted immunostaining pattern. The presence of γH2AX can critically distinguish between TUNEL-positive apoptotic DNA ladders and chromosomal breaks [27], supporting that tubular cell death associated with an γH2AX-positive signal occurred in early *inv* DC cystogenesis. This type of cell death mechanism might be fundamentally conserved for epithelial homeostasis as recently reported in gut tissue [28]. On the other hand, γH2AX was reportedly involved in homeostatic suppression of proliferation by restricting stem cell proliferation without obvious DNA damage [39, 40]. The role of such events in our cystic model was not addressed in the context of DNA damage. Further investigation is needed to elucidate whether the biological roles of γH2AX in tubular cell death are protective or progressive in the *inv*DC cystic model.

### How tubular cell loss contributed to renal cytogenesis in the *inv*DC model?

Planar cell polarity (PCP) is the coordinated orientation of tissue cells in an orthogonal direction to apico-basal polarity [41]. Renal tubular cells have PCP, controlled by oriented cell division (OCD), within the closed epithelial tubules and a defect in PCP was commonly proposed as a cellular phenotype in PKD [15] [16]. On the other hand, it is controversial whether loss of OCD occurs before or after renal cystogenesis [42] [43]. In addition, it has been shown that core PCP signaling regulated renal tubular diameter but loss of this control did not induce renal cysts [44]. Although, the *inv* mutation was also associated with PCP-associated genes such as disheveled [45], their relationship to renal cystogenesis was uncleared in *inv* mutant model. Remarkably, we observed early *inv*DC tubular phenotype was very similar to the morphological features of acute tubular necrosis in the kidney, with a flattened tubular morphology, sloughing cells and luminal necrotic debris [46]. We hypothesized that tubular loss of proliferating cells by renal phagocytosis triggered a defective tubular reorganization, including a flattened tubular morphology. Thus, the tubular dilatation proceeded in precystic tubules. This effect accumulated over time, causing a mitotic angle defect and leading to renal cyst expansion in the *inv*DC mutants. The concept that tubular cell loss is critical in renal cystogenesis is compatible, at a molecular level, with a recent report that the C-terminus of the *inv* protein interacted with Akt signaling, an important process for cell survival [6]. To investigate our hypothesis, a live imaging approach would be required although it would be technically quite difficult to observe kidney development under proper conditions for the long time periods needed. Recent technical breakthroughs, enabling production and maintenance of nephrogenic progenitor cells like the ES cell line [47], might be useful to observe *in vitro* tubular formation in future research.

### Renal tubular cell kinetics elucidated in a multi-population shift model

In multicellular organisms, several cell populations become specialized within organs and tissues to perform functions and specific roles, as in the hematopoietic stem cell system. However, in some other organs and tissues, including the kidney, it is not clear how many cell populations contribute to formation of tubular structure leading to terminal differentiation. Our model revealed that a restricted tubular cell population contributed to cortical tubular formation and identified a progenitor-like rapid cell cycling population. This helped increase understanding of the complex cellular phenotypes in *inv*DC cystogenesis, when fitted with the experimental data from P9 to P30. However, our model could did not predict the higher proliferation rates from P4 to P9 (average ratio at P9/P4 = 3.4). Because nephrogenesis by non-tubular cells is believed to continue at P4, the shorter doubling time or loss of a slow cell cycling population (SP) would be assumed. Further double labeling experiments and model customization in future studies will be required to more fully understand the cellular kinetics.

Although many cell proliferation models have been described, to our knowledge all were consistent with a single cell population and most were focused on single cancer cell lines, not multiple cell populations. In addition, it is difficult to understand proliferation dynamics using only an experimental approach when considering multiple cell populations and the mechanical principles of proliferation control in multiple cell populations are not understood. In our modeling study, we observed some interesting examples of simulation of proliferation dynamics. From these simulations, we also found that general declines in cell proliferation curve could be explained by increased a non-dividing cell population (NDP) in addition to cellular loss, that is, our model predicted that the NDP (38%) emerged under normal cortical tubular development. This numerically confirmed the biological significance of both a quiescent cell population and a longer slow cell cycling one in size control of kidney, potentially because quiescent cells may reenter into the cell cycling at certain times. Although significant increase of a third SP (doubling time = 9 d) in mutants might be explained by the decreased probability (*r*, 0.60 → 0.55) of cell division in SP, the difference in ratios of NDP (1-*r*) between controls (*r* = 0.60) and mutants (*r* = 0.55) was approximately 2.0% (Fig 3c right graph). This indicated that the quiescent cell population size was not dramatically changed in the *inv*DC mutant, compared with in controls. Considering this, tubular cell loss (*q*2 = 0.12: 9.6% in total) could be identified as a critical phenotype in early *inv*DC cystogenesis. Given that tubular cell loss is linked to mitotic cell death and then rapidly cleared by renal phagocytosis, an indicative value of cell death as shown in Fig 5c must be underestimated. Future studies using live imaging analysis of renal tubular development, overcoming the technical hurdles, might further elucidate the involvement of cell death and the associated mechanisms.

## Conclusions

In conclusion, tubular cell loss was identified as an early cystogenic phenotype in the *inv*DC kidney model. We concluded that total tubular cell numbers were unaffected in early cystogenesis in the *inv*DC kidney because of tubular loss of the proliferating cells, suggesting that tubular cell loss is critical in both early renal cystogenesis and tubular homeostasis. The underlying cellular kinetics in early cortical cystogenesis were unveiled by a new multi-population shift model, not only providing new insights into the *inv*DC mutant phenotype but also suggesting that a few restricted tubular cell populations contribute to postnatal cortical tubular formation.

## Materials and methods

### Animals

FVB/N and transgenic *inv*/*inv* mice carrying *inv*ΔC:: GFP (*inv*ΔC) were maintained as previously descried [7] [8] in a pathogen-free state at the animal facility of Kyoto Prefectural University of Medicine, Japan. All experimental procedures were approved by the Committee for Animal Research, Kyoto Prefectural University of Medicine. We used +/*inv* or +/+ mice carrying the *inv*ΔC transgene as wild-type controls for all experiments.

### Quantification of whole kidney genomic DNA

Whole kidney gDNA was quantified using DNA zol Genomic DNA isolation reagent (Molecular Research). Whole kidney weight at each developmental stage was measured, and gDNA was isolated according to the manufacturer’s instructions. Over six kidneys were evaluated from mice in each group (control and *inv*DC mutant). The concentration of gDNA was measured with a BioPhotometer plus (Eppendorf), and the total amount of DNA was calculated.

### Immunohistochemistry

Isolated kidneys were fixed in 4% paraformaldehyde in phosphate-buffered saline (PBS) according to standard procedures. Kidneys were cryoprotected with 30% sucrose in PBS and embedded in optimal cutting temperature compound (Tissue-Tek, Miles; Elkhart, IN, USA), and then frozen in liquid nitrogen and stored at −80°C until sectioning. Kidney sections (10-μm thick) were immunostained according to a standard protocol. Antibodies and chemicals used for immunohistochemistry were as follows: mouse BrdU (Sigma), rabbit polyclonal phosphor-histone H3 (Ser10) (Millipore), rabbit polyclonal γH2AX (Ser139) (Cell Signaling, Danvers, MA), Alexa Fluor 633 phalloidin (Thermo Fisher Scientific), 4’6’ diamino-2-phenylindole (DAPI) solution (Dojindo, JAPAN), fluorescein isothiocyanate-coupled *Lotus tetragonolobus* lectin (LTL) (Vector Laboratories, Burlingame, CA, USA), Alexa Fluor 488- or 555-conjugated goat anti-mouse or rabbit secondary antibodies (Invitrogen).

### Detection of apoptosis

Apoptosis was measured using a terminal deoxynucleotidyl transferase dUTP nick-end labeling (TUNEL) assay with an *In situ* Apoptosis Detection Kit (TAKARA, JAPAN) according to the manufacturer’s instructions, followed by DAPI staining. Renal cortical sections from control and *inv*DC mutant mice at P9 and P15 were used for the TUNEL assay (n = 10 fields with 40× objective/age). The staining reaction without the transferase was used as the negative control, and DNase I-treated slides were used as positive controls. For evaluation of renal apoptosis as another positive control, mice were sacrificed at 2 and 3 days after a single intraperitoneal injection of PBS or the nephrotoxic drug, cisplatin, at 20 mg/ml/kg (Wako, JAPAN). The stained slides were analyzed by LSM510 confocal microscopy (Zeiss, Germany) and TUNEL-positive tubular cell number was counted per area.

### EdU-BrdU double labeling

Tubular cell proliferation kinetics were evaluated by both bromodeoxyuridine (BrdU) and 5-ethynyl-2'-deoxyuridine (EdU) incorporation. First, mice were injected intraperitoneally with a solution of PBS containing EdU (Click-iT EdU Alexa Fluor 647 Imaging kit, Thermo Fisher scientific) according to the manufacturer’s instructions at 24 h before the isolation of kidneys. Next, a BrdU solution (10 mg/kg) was injected at 3 h before the isolation of kidneys. Kidneys were fixed and visualized as described above. For detection of BrdU signals, sections were treated with 1.5 M hydrochloric acid at 37°C for 20 min before other immunostaining procedures. Microscopic fields (40×) of renal sections were randomly selected and processed for immunostaining as mentioned above. After counterstaining with DAPI, all specimens were imaged by LSM510 confocal microscopy (Zeiss, Germany). The number of EdU- or BrdU-positive nuclei and the total tubular cells were counted and then accumulated from all images with a Zeiss LSM Image Browser (Zeiss, Germany). The percentages of EdU- or BrdU-positive tubular cells were calculated by dividing the cell numbers by total tubular cell numbers and then represented as double proliferation indexes. The doubling time of cortical tubular cells was evaluated based on the percentages of the EdU/BrdU-double positive cells referring to previous report [17]. Briefly, EdU was intraperitoneally injected into the mice at P8, followed by multiple BrdU injections at 6 h and 12 h before harvesting kidneys at P11, P14, and P17. The percentages of EdU/BrdU-double positive or EdU-positive tubular cells per total tubular cells was respectively calculated as described above and compared between control and *inv*DC mutant mice at the indicated postnatal mice age (n = 3).

### Renal tubular characterization

For the comparison of average tubular cell number per area of renal cortex in P15 mice, confocal imaging was performed in randomly selected fields of renal cortex (20 fields from two mice in each group) by using an LSM510 confocal microscope (Zeiss, Germany). We first identified tubules which were mostly observed as the circular or elliptical morphology with F-actin staining in renal cortex. All tubules identified on acquired images were numbered with tubular diameters (both major and minor axis) which were manually measured using the LSM Image Browser. The number of tubular cells per each imaging fields were counted based on the nuclear staining as mentioned above. The average tubular cell number per cortical area (150 μm^2^ with a 40× objective) was calculated by dividing the accumulated tubular cell number by the total field number (n = 20).

For evaluation of the relationship between tubular cell number and diameter, the cross-sectional tubule was defined with a major/longer to minor/shorter axis ratio ≤1.5 as previously described [48]. Those tubules that appeared to be morphologically almost round shape were selected for the counting. Then, the numbers of epithelial cells per tubular cross-section were counted and the percentages of tubules having the given numbers of cells were calculated by dividing the tubule numbers by total tubule numbers. The diameter per tubular cross-section was defined by selecting the minor diameter and then compared between control and mutant. For TUNEL-negative cell death evaluation, luminal debris was counted as DAPI-nuclear staining with small-irregular or dot-like debris in tubular lumens or on the luminal side of tubular cells. LTL-staining was performed for clear identification of luminal nuclear debris or morphology of cells with nuclear protrusions.

### Mathematical stochastic model for proliferation

Classically, stochastic models for proliferation are used to analyze cancer initiation or expansion, based on a single population [49] [50]. However, we found that at least two types of doubling time populations were maintained during tubular development (Fig 3b), as supported by previous observations [17]. Therefore, we used a multipopulation shift model (Fig 4b) consisting of a rapid cell cycling population (RP) and a slow cell cycling population (SP). The SP was most likely to be derived from the RP during tubular development. This can be explained as follows: a population shift from a RP to a SP is a change in cell fate resulting in asymmetrical mitosis. Furthermore, the cellular loss probability (*q*) was combined as implied in Fig 3c, although tubular cell loss is considered mostly to not occur during normal tubular development. A non-dividing cell population (NDP), as a quiescent population or a longer slow cell cycling population, is generally believed to be emerged during a biological development. For this concept, the probability of cell division (*r*) was added to the model. Therefore, the mixed growth dynamics of the two populations over time (*t*) can mostly be represented by the following equations:

$$N(t)=N_{1}(t)+N_{2}(t)$$

$${N_{1}(t)=2(1-q_{1})(1-p)}^{\zeta (t)}N_{0}$$

$$N_{2}(t)=\sum_{i}^{\zeta (t)}\frac{p}{1-p}{\left(2r(1-q_{2})+(1-r)\right)}^{\eta (t,i)}{\left(2(1-q_{1})(1-p)\right)}^{i}N_{0}$$

$$\zeta \left(t\right)=\left[\frac{24t}{T_{1}}\right]$$

$$\eta \left(t,i\right)=\left[\frac{24t-T_{1}i}{T_{2}}\right]$$

where N1(*t*) or N2 (*t*) and N0 (*t*) are the cell number of the RP or SP at time *t* and time *zero*, respectively; *p* is the probability of shift from a RP to a SP; and *q* denotes the probability of cellular loss. *T1* and *T2* are the cell cycle length of the RP and SP, respectively, defined as 48 h and 168 h in the current study based on the present data and the related reference [17]. All other parameters are defined in Table 2. The model underlying equations were written with text editor, ATOM (https://atom.io/) and we simulated the stochastic models by using the Python 3.6.3 software program (https://www.python.org/downloads/) coupled with ATOM. We also utilized the Python packages, the numerical package Numpy-1.7.0 (https://pypi.python.org/pypi/numpy/1.7.0) and plotting package, Matplotlib-2.1.0 (https://pypi.python.org/pypi/matplotlib) for the graph presentation of simulation results.

**Table 2:** Definition of mathematical parameters for the multiple proliferation model.

Parameter	Description
***T1***	**Cell cycle time of a rapid cell cycling population (RP) (hour)**
***T2***	**Cell cycle time of a slow cell cycling population (SP) (hour)**
***P***	**Population shift probability from RP to SP**
***q1***	**Cell loss probability of RP during or after the cell division**
***q2***	**Cell loss probability of SP during or after the cell division**
***r***	**Probability of cell division in SP at T2**
***N0***	**Initial cell number of RP**
***t***	**Time (day)**

### Statistical analyses

We evaluated differences between the experimental groups with the Kolmogorov-Smirnov test, unpaired t-test, Fisher’s chi-squared test, and Mann-Whitney *U* test as indicated in each figure legend. Data are presented as mean ± standard error of the mean (SEM). A value of *P* < 0.05 was considered statistically significant. All statistical analyses were performed using Graph Pad Prism 7 (Graph Pad Software Inc., La Jolla, CA, USA).

## Supporting information

S1 Fig Almost no BrdU was incorporated in tubular cells in the renal cortex at P4, consistent with no cortical growth during nephrogenic cessation [51]. **(a)** Experimental scheme shows that EdU was intraperitoneally injected into control mice at P3, and BrdU was administrated at 3 h before the isolation of kidneys at P4, P5, and P6. **(b)** Confocal images of the renal cortex with EdU (cyan), BrdU (magenta), LTL (green), and DAPI (blue) staining at P4, P5, and P6. The EdU-labeled cells contributed to both nephrogenic pretubular aggregates and the derived newly differentiated tubules at P4 (yellow arrows), while the tubular contribution was increased from P5 with BrdU-labeling (white arrows). **(c)** The ratio of EdU- or BrdU-positive tubular cells per total number of cells is presented. Scale bar, 20 μm.
(TIF)

S2 Fig Significant differences in whole kidney gDNA contents in control and mutant mice at later stage of cystogenesis. The experiment was examined at P30 as described in Material and Methods. The Mann-Whitney U test was used with *P < 0.05 (± standard error of the mean (SEM)).
(TIF)

S3 Fig There was no significant difference in the length of S phase in control and *inv*DC tubular cells. **(a)** Experimental scheme for evaluation of S phase exit timing. EdU was intraperitoneally injected into mice at 6 h, 9 h, or 12 h before P15 kidney sampling. BrdU was then injected in each EdU-treated mouse before kidney sampling. Immunohistological sectioning and analysis was performed for counting EdU-BrdU double-positive cells as described above. **(b)** The EdU-BrdU double-labeling cell ratio per total EdU-positive tubular cells from three mice at each indicated time was analyzed with Fisher’s Chi-square distribution test (*P < 0.05), indicating that both control and *inv*DC mutant tubular cells exited from S phase with the same timing within about 9 h.
(TIF)

S4 Fig Apoptosis was not detected in early *inv*DC cystogenesis. **(a)** Positive control confirmation of TUNEL assay with or without the transferase in the presence of DNase I treatment. Adult normal kidney sections at P30 were processed according to the manufacturer’s instructions of the TUNEL assay kit. Scale bar, 20 μm. **(b)** Positive detection of cisplatin-induced renal apoptosis in the normal renal cortex. Kidney sections at 48 h and 72 h following intraperitoneal injection with cisplatin. **(c)** TUNEL-positive tubular cells with condensed or fragmented apoptotic nuclei. Images represent magnified images of cells indicated by arrows in **(b)**. **(d)** Assessment of TUNEL-positive cells in renal cortical sections. Data are presented as mean ± SD per 10 fields at each hour. Normal adult mice at P30 were used for the positive control experiment. Scale bar, 50 μm. Controls are not shown.
(TIF)
