# Supplementary figures and images for: Tubular cell loss in early inv/nphp2 mutant kidneys represents a possible homeostatic mechanism in cortical tubular formation
Source: PLoS One. 2018 Jun 11;13(6):e0198580. doi: 10.1371/journal.pone.0198580 (PMC5995398; doi:10.1371/journal.pone.0198580)

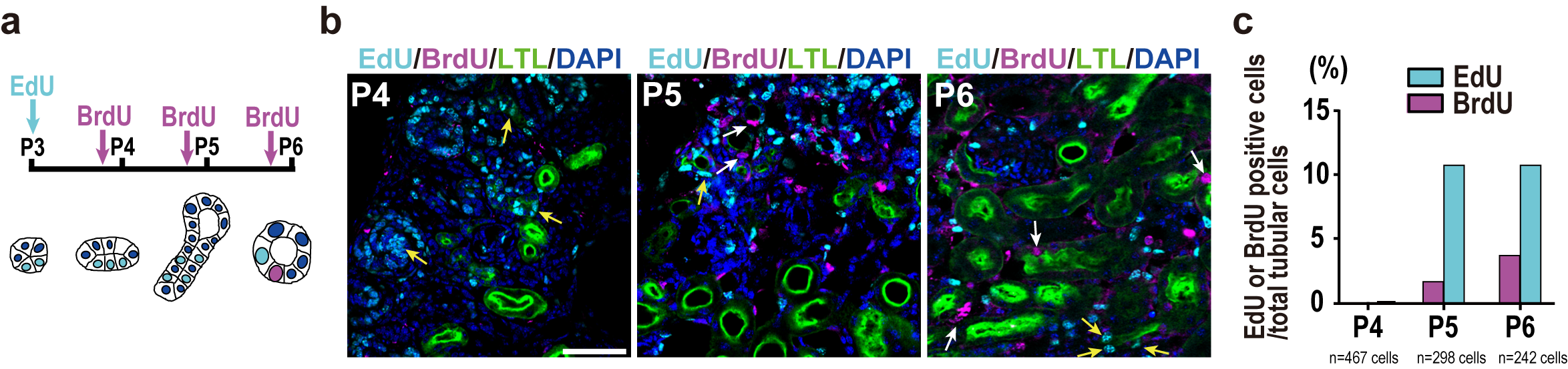

Supplement: S1 Fig — (a) Experimental scheme shows that EdU was intraperitoneally injected into control mice at P3, and BrdU was administrated at 3 h before the isolation of kidneys at P4, P5, and P6. (b) Confocal images of the renal cortex with EdU (cyan), BrdU (magenta), LTL (green), and DAPI (blue) staining at P4, P5, and P6. The EdU-labeled cells contributed to both nephrogenic pretubular aggregates and the derived newly differentiated tubules at P4 (yellow arrows), while the tubular contribution was increased from P5 with BrdU-labeling (white arrows). (c) The ratio of EdU- or BrdU-positive tubular cells per total number of cells is presented. Scale bar, 20 μm. (TIF) [file pone.0198580.s001.tif]

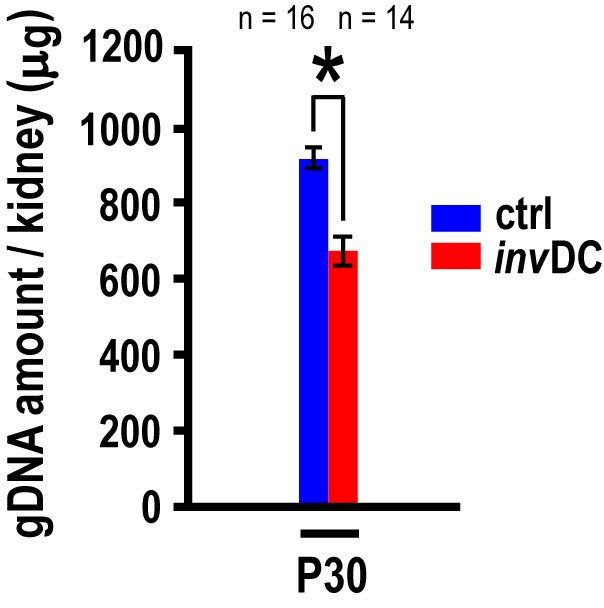

Supplement: S2 Fig — The experiment was examined at P30 as described in Material and Methods. The Mann-Whitney U test was used with *P < 0.05 (± standard error of the mean (SEM)). (TIF) [file pone.0198580.s002.tif]

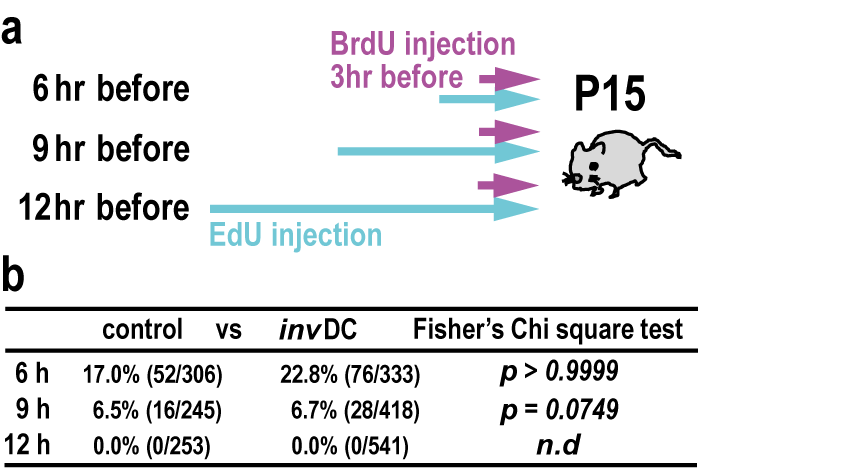

Supplement: S3 Fig — (a) Experimental scheme for evaluation of S phase exit timing. EdU was intraperitoneally injected into mice at 6 h, 9 h, or 12 h before P15 kidney sampling. BrdU was then injected in each EdU-treated mouse before kidney sampling. Immunohistological sectioning and analysis was performed for counting EdU-BrdU double-positive cells as described above. (b) The EdU-BrdU double-labeling cell ratio per total EdU-positive tubular cells from three mice at each indicated time was analyzed with Fisher’s Chi-square distribution test (*P < 0.05), indicating that both control and invDC mutant tubular cells exited from S phase with the same timing within about 9 h. (TIF) [file pone.0198580.s003.tif]

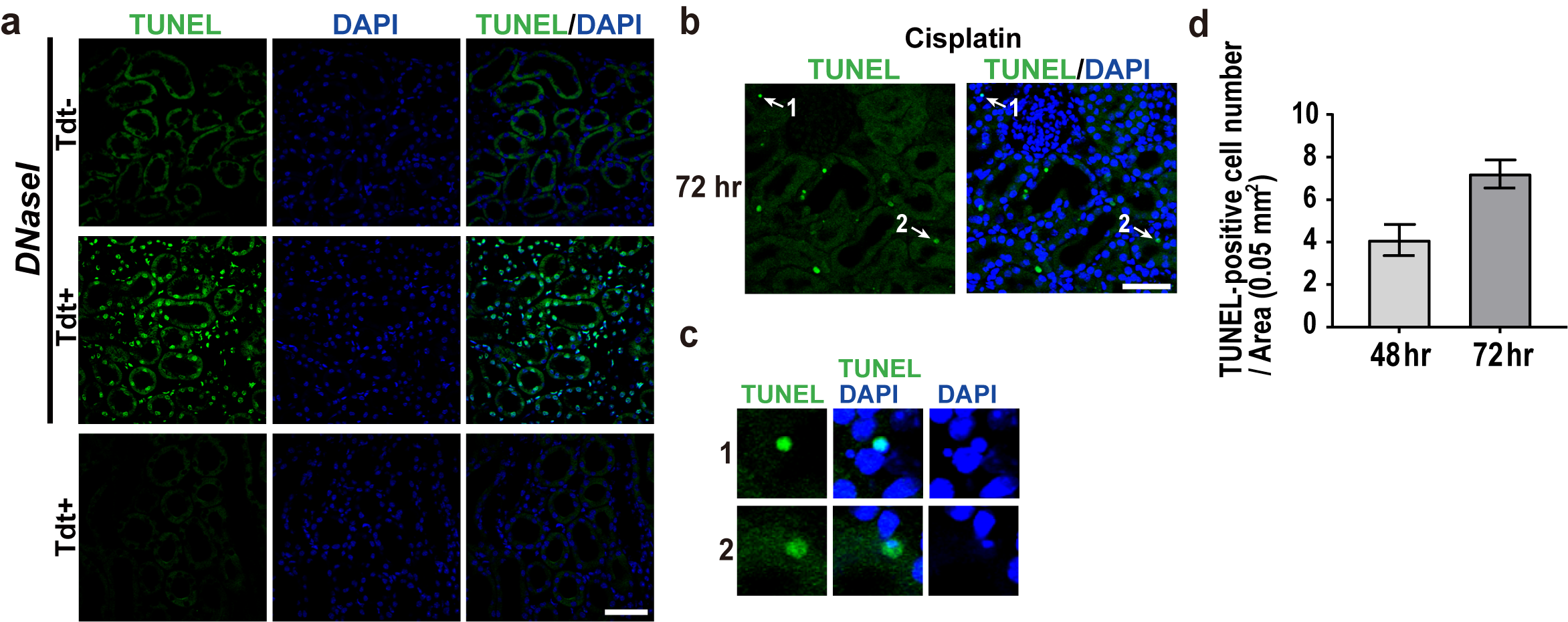

Supplement: S4 Fig — (a) Positive control confirmation of TUNEL assay with or without the transferase in the presence of DNase I treatment. Adult normal kidney sections at P30 were processed according to the manufacturer’s instructions of the TUNEL assay kit. Scale bar, 20 μm. (b) Positive detection of cisplatin-induced renal apoptosis in the normal renal cortex. Kidney sections at 48 h and 72 h following intraperitoneal injection with cisplatin. (c) TUNEL-positive tubular cells with condensed or fragmented apoptotic nuclei. Images represent magnified images of cells indicated by arrows in (b). (d) Assessment of TUNEL-positive cells in renal cortical sections. Data are presented as mean ± SD per 10 fields at each hour. Normal adult mice at P30 were used for the positive control experiment. Scale bar, 50 μm. Controls are not shown. (TIF) [file pone.0198580.s004.tif]
